# Fabrication of Multiferroic Co-Substituted BiFeO_3_ Epitaxial Films on SrTiO_3_ (100) Substrates by Radio Frequency Magnetron Sputtering

**DOI:** 10.3390/ma4061087

**Published:** 2011-06-09

**Authors:** Husne Ara Begum, Hiroshi Naganuma, Mikihiko Oogane, Yasuo Ando

**Affiliations:** Department of Applied Physics, Graduate School of Engineering, Tohoku University, 6-6-05, Aza-aoba, Aoba-ku, Sendai 980-8579, Japan; E-Mails: begum@mlab.apph.tohoku.ac.jp (H.A.B.); oogane@mlab.apph.tohoku.ac.jp (M.O.); Ando@mlab.apph.tohoku.ac.jp (Y.A.)

**Keywords:** multiferroic, BiFeO_3_, BiCoO_3_, r.f. magnetron sputtering

## Abstract

The 10 at.% Co-substituted BiFeO_3_ films (of thickness 50 nm) were successfully prepared by radio frequency (r.f.) magnetron sputtering on SrTiO_3_ (100) substrates with epitaxial relationships of [001](001)Co-BiFeO_3_//[001](001)SrTiO_3_. In this study, a single phase Co-substituted BiFeO_3_ epitaxial film was fabricated by r.f. magnetron sputtering. Sputtering conditions such as Ar, O_2_ gas pressure, annealing temperature, annealing atmosphere, and sputtering power were systematically changed. It was observed that a low Ar gas pressure and low sputtering power is necessary to suppress the formation of the secondary phases of BiO*_x_*. The Co-substituted BiFeO_3_ films were crystalized with post-annealing at 600 °C in air. The process window for single phase films is narrower than that for pure BiFeO_3_ epitaxial films. By substituting Fe with Co in BiFeO_3_, the magnetization at room temperature increased to 20 emu/cm^3^. This result suggests that Co-substituted BiFeO_3_ films can be used in spin-filter devices.

## 1. Introduction

**Figure 1:**
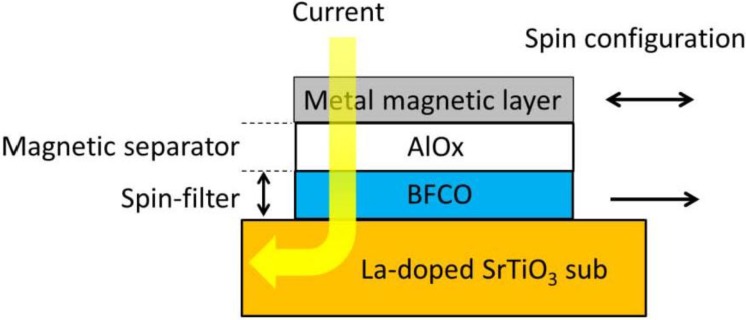
Schematic illustration of stacking structure of spin-filter device using Bi(Fe_0.9_Co_0.1_)O_3_.

Recently, Bi-based multiferroic materials have been attracting considerable attention because of their varied applications. BiFeO_3_ is a well-known example of multiferroic material with a rhombohedrally distorted perovskite structure (space group: *R*3*c*). Displaying ferroelectric properties, BiFeO_3_ has a high transition temperature (*T*_C_) of 1100 K [1] and exhibits very large spontaneous polarization (100–150 µC/cm^2^) at room temperature (RT) [2,3]. With regard to magnetism, the magnetic transition temperature of BiFeO_3_ is above RT (*T*_N_ = 653 K) [4]; however, it is antiferromagnetic and has a *G*-type spin configuration. In this configuration, the nearest neighboring Fe moments are aligned antiparallel to each other, and there is a six-fold degeneracy resulting in an effective “easy magnetization plane” for the orientation of the magnetic moments within the (111) plane. Owing to the Dzyaloshinskii-Moriya (DM) interaction, the symmetry allows for canting of the antiferromagnetic sublattices resulting in weak local spontaneous magnetization [5], which is macroscopically canceled by the spiral spin structure in which the antiferromagnetic axis rotates through the crystal with an incommensurate long-wavelength period of 62 nm. This spiral spin structure might be suppressed in film form and magnetic moment due to a weak ferromagnetism of ~0.1 μ_B_/Fe atom being observed. However, this small magnetic moment is not suitable for applications such as spintronics because weak magnetic moments and spin-filter effects are difficult to detect using a magnetic sensor. Recently, it was proposed that substitution of iron atoms at the *B* sites with other 3*d* transition atoms, such as cobalt, would result in a local ferrimagnetic spin configuration or the collapse of the spiral spin structure leading to macroscopic magnetization. Indeed, the magnetic moment has been reported to increase when the Fe atom in BiFeO_3_ is replaced with Mn or Co atom [6,7,8]. As a result, these materials are in growing demand for use in spintronics applications. In addition, magnetization increases with increasing Co substitution in the rhombohedral structure [9]; the crystal symmetry changes from rhombohedral to tetragonal at 15–20 at.% of Co substitution [10,11]. Based on this information, in this study, 10 at.% Co-substituted BiFeO_3_ was used. When used for spintronics applications such as multivalued memory that uses a spin-filter structure, a few-nanometer-thick high-quality film, a flat surface/interface, and good compatibility with the thin metal film process are required. The actual spin-filter structure designed by us consists of conductive La doped SrTiO_3_ sub./Co-substituted BiFeO_3_/AlO_x_/metal magnetic layer/cap layer. The Co-substituted BiFeO_3_ layer was prepared by heat treatment for crystalizing perovskite structure and AlO*_x_* and the metal magnetic layer were deposited at ambient temperature to exclude interfacial oxidation. The schematic illustration of stacking structure of spin-filter device is shown in Figure 1. Various methods for preparing Co-substituted BiFeO_3_ epitaxial films, such as metalorganic chemical vapor deposition (MOCVD) [10] and chemical solution deposition (CSD) [11], have been reported. However, these chemical processes are not suitable for metal processes due to carbon impurities. One of the promising methods for preparing both oxides and metals layers are radio frequency (r.f.) sputtering [12]. However, there is no report of Co-substituted BiFeO_3_ thin epitaxial films fabricated by r.f. magnetron sputtering. The aim in this study is to determine the preparation conditions of single phase Co-substituted BiFeO_3_ [Bi(Fe_0.9_Co_0.1_)O_3_] epitaxial films on SrTiO_3_ (100) substrates by conventional r.f. magnetron sputtering.

## 2. Experimental Procedure

The Co-substituted BiFeO_3_ films having a thickness of 50 nm were fabricated by r.f. magnetron sputtering on single crystal SrTiO_3_ (100) substrates. The base pressure was below 8 × 10^−^^5^ Pa. The sputtering target of Bi(Fe_0.9_Co_0.1_)O*_x_* consisted of Bi_2_O_3_ and Fe_2_O_3_ powder that had been sintered in air. The size of disc-shape target was 0.076 m in diameter. The sputtering conditions such as argon (Ar) and/or oxygen (O_2_) gas pressure, sputtering power, heating temperature in vacuum or air were systematically changed to fabricate single phase Bi(Fe_0.9_Co_0.1_)O_3_ films. The structure of the films was characterized by a conventional θ/2θ X-ray diffraction (XRD, Cu-K_α_) pattern, and the surface morphology was investigated by atomic force microscopy (AFM). The homogeneity of the composition of the film surface was observed by field-emission scanning electron microscopy (FE-SEM). The magnetic properties at RT were measured by a superconducting quantum interference device (SQUID) magnetometer.

## 3. Results and Discussion

**Figure 2:**
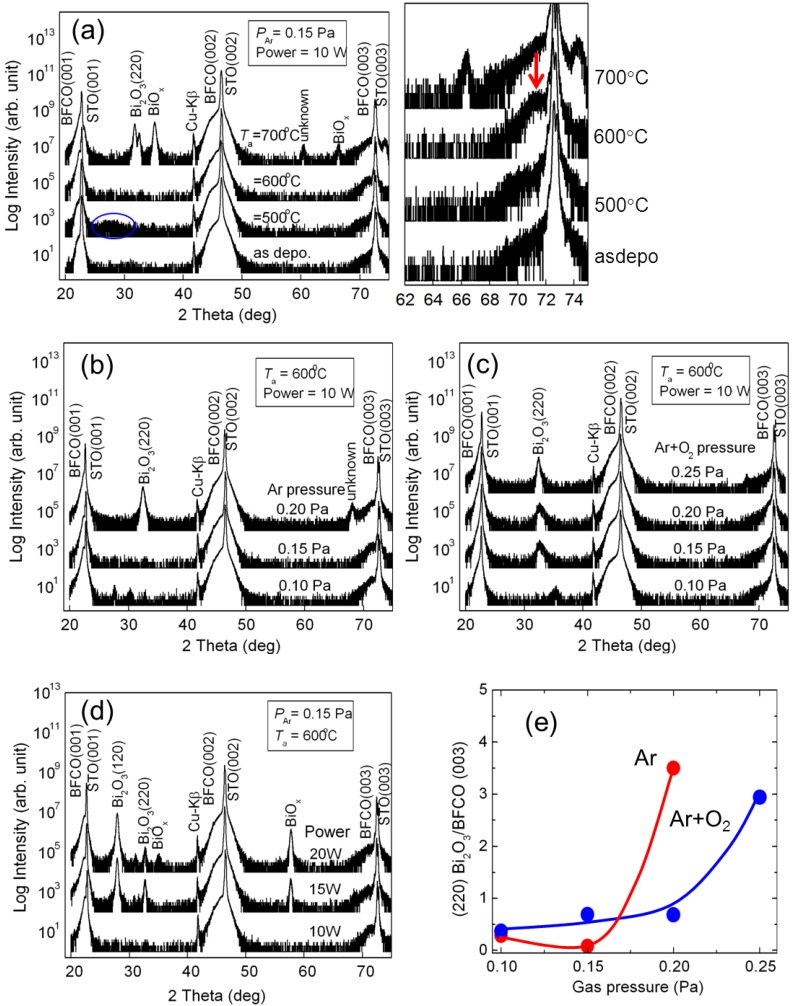
(**a**) X-ray diffraction (XRD) patterns for the Bi(Fe_0.9_Co_0.1_)O_3_ films annealed at different temperatures, (**b**) and (**c**) Ar and Ar + O_2_ (3:1) gas pressure dependence during sputtering, (**d**) Input power dependences of the XRD patterns at Ar gas pressure of 0.15 Pa annealed at 600 °C, and (e) Ar and Ar + O_2_ gas pressure dependence of the XRD peak intensity ratio of (200) Bi_2_O_3_/(003) Bi(Fe_0.9_Co_0.1_)O_3_. A single phase Bi(Fe_0.9_Co_0.1_)O_3_ film was formed with low Ar gas pressure and annealed at 600°C in air.

The films deposited by r.f. magnetron sputtering using Ar and Ar + O_2_ gases at an ambient temperature had an amorphous and/or nanocrystalline structure. In order to crystallize the perovskite structure of Bi(Fe_0.9_Co_0.1_)O_3_ films, the samples were annealed under both vacuum and air conditions at various temperatures. In the case of the samples annealed under vacuum conditions, secondary phases such as BiO*_x_* were formed; therefore, the experimental data of the vacuum condition is not discussed in this paper. Figure 2(a) shows the annealing temperature dependence of the θ/2θ XRD patterns for the Bi(Fe_0.9_Co_0.1_)O_3_ films annealed in air. The Ar gas was used for sputtering. The formation of the perovskite structure in XRD patterns was identified by the high angle peak of (003) Bi(Fe_0.9_Co_0.1_)O_3_, because the lattice mismatch decreased with increasing Co content. The films crystallized above the annealing temperature of 500 °C with a broad unknown peak of 2θ = 27° (indicated as blue-circle in Figure 2(a)); however, the perovskite phase was not observed at 500 °C. After annealing at 600 °C, only the perovskite peaks of (003) Bi(Fe_0.9_Co_0.1_)O_3_ was observed near the peaks of the (003) SrTiO_3_ substrate. [Figure 2(a)] At 700 °C, the secondary phases of BiO*_x_* were observed, and the peak of the perovskite structure became weak. The dependence of phase formation on annealing temperature is almost the same as that of the CSD process [13]; however; it is lower than that of the MOCVD method [10]. 

The influence of a variety of gases and gas pressures on the formation of single phase Bi(Fe_0.9_Co_0.1_)O_3_ films was also investigated. Figure 2(b) and (c) show the Ar and Ar + O_2_ gas pressure dependence of the XRD patterns. The annealing temperature was fixed at 600 °C in air because BiO_x_ of secondary phase has a tendency to suppress at 600 °C. Single phase Bi(Fe_0.9_Co_0.1_)O_3_ films formed at low Ar gas pressure, and secondary phases of (220) Bi_2_O_3_ formed at 2θ = 32° when the gas pressure increased to 0.20 Pa. This tendency of gas pressure is roughly consistent with the case of BiFeO_3_ films [14,15,16]. In the case of Ar + O_2_ gas, secondary phases of (220) Bi_2_O_3_ formed for all gas pressures [Figure 2(c)]. Figure 2(d) shows the input power dependence of the XRD patterns with an Ar gas pressure of 0.15 Pa annealed at 600 °C. It was observed that the secondary phases of BiO*_x_* increased with increasing input power. Therefore, low sputtering power is needed to obtain single phase Bi(Fe_0.9_Co_0.1_)O_3_. Figure 2(e) shows the Ar and Ar + O_2_ gas pressure dependence of the XRD peak intensity ratio of (220) Bi_2_O_3_/ (003) Bi(Fe_0.9_Co_0.1_)O_3_. For an Ar + O_2_ gas pressure of 0.10 Pa [Figure 2(c)], (220) Bi_2_O_3_ was not observed; therefore, an unknown XRD peak at 2θ = 35° was obtained as the secondary phase. The quantity of the secondary phase of Bi_2_O_3_ has a tendency to increase with increasing gas pressure. The sputtering conditions and optimal condition for obtaining single phase of Bi(Fe_0.9_Co_0.1_)O_3_ film was summarized in Table 1. From these results, it can be considered that the formation of single phase Bi(Fe_0.9_Co_0.1_)O_3_ films was sensitive to preparation processes; the single phase formed at relatively low Ar gas pressure, low sputtering power, and post-annealing at 600 °C in air.

**Table 1:** Sputtering parameters and optimal conditions for preparing single phase of Bi(Fe_0.9_Co_0.1_)O_3_. The symbol (*) indicates the parameter in which the single phase was observed.

Temperature (°C)	Ar+O_2_ gas pressure (Pa)	Ar gas pressure (Pa)	Input Power (W)
as-depo.	0.10	0.10	10*
500	0.15	0.15*	15
600*	0.20	0.20	20
700	0.25		

**Figure 3:**
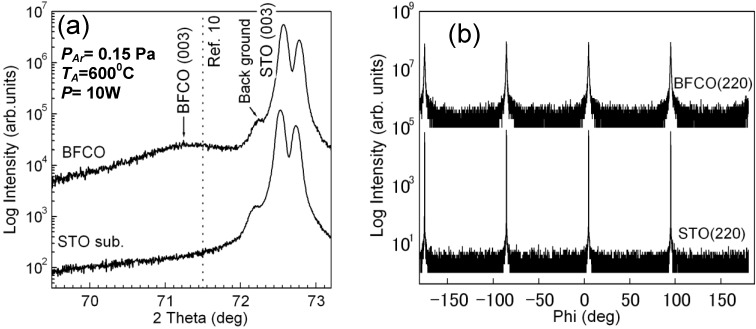
(**a**) XRD profile around Bi(Fe_0.9_Co_0.1_)O_3_ (300). The peak position of Bi(Fe_0.9_Co_0.1_)O_3_ prepared by CSD method is indicated by the dotted line [11]; (**b**) phi-scan measurement at Psi = 45° using the BiFeO_3_ (202) and SrTiO_3_ (202) peaks.

We confirmed that the single phase Bi(Fe_0.9_Co_0.1_)O_3_ film prepared at an Ar gas pressure of 0.15 Pa and annealed at 600 °C in air contained an epitaxial structure. Figure 3(a) shows the XRD profiles magnified around SrTiO_3_ (003). The peak position of Bi(Fe_0.9_Co_0.1_)O_3_ prepared by the CSD method is indicated as a dotted line [11]. The XRD peak position of Bi(Fe_0.9_Co_0.1_)O_3_ (003) was close to that previously reported, and the lattice parameter of a = 0.3948 nm in Bi(Fe_0.9_Co_0.1_)O_3_ film was almost the same as that of Bi(Fe_0.9_Co_0.1_)O_3_ film prepared by CSD method (a = 0.394 nm [11]). However, the peak intensity is relatively weak. Figure 3(b) shows the phi-scan measurement at Psi = 45° using the BiFeO_3_ (202) and SrTiO_3_ (202) peaks. We did not change sample configuration against beam line when measuring BiFeO_3_ and SrTiO_3_ layer. The peaks of Bi(Fe_0.9_Co_0.1_)O_3_ were observed to be the same as that of SrTiO_3_ substrate; this indicates that the Bi(Fe_0.9_Co_0.1_)O_3_ films were epitaxially grown on the SrTiO_3_ substrate with cube-on-cube: [001](001) Bi(Fe_0.9_Co_0.1_)O_3_//[001](001) SrTiO_3_.

**Figure 4:**
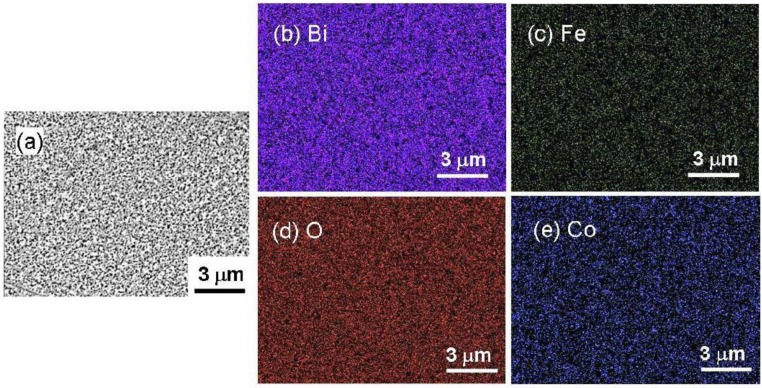
(**a**) SEM image, and elementary mapping images of (**b**) Bi, (**c**) Fe, (**d**) O, and (**e**) Co using SEM-EDX for single phase Bi(Fe_0.9_Co_0.1_)O_3_ epitaxial films.

The composition of Bi(Fe_0.9_Co_0.1_)O_3_ film prepared at an Ar gas pressure of 0.15 Pa and annealed at 600 °C was confirmed by plan-view FE-SEM and SEM-EDX. Figure 4 shows SEM image, and elementary mapping images of Bi, Fe, Co, and O, respectively, captured by SEM-EDX. The elemental mapping for each element did not show obvious agglomeration of elements, indicating that the larger impurity grains were not formed on surface of film. However, further investigation of smaller grains around the size of superparamagnetic limit of Co should be confirmed by transmission electron microscopy (TEM) observation.

**Figure 5:**
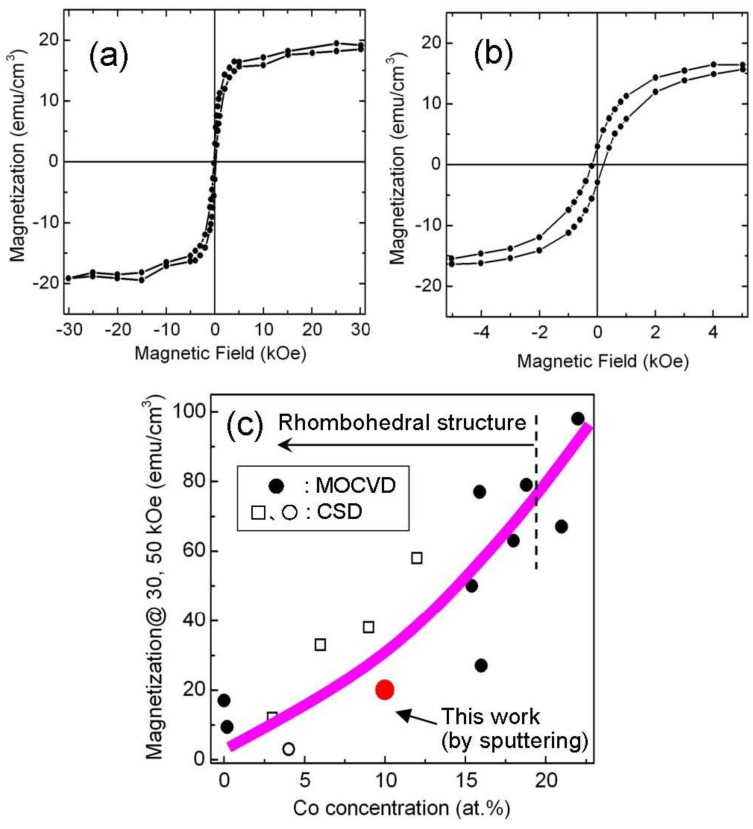
(**a**) In-plane *M*-*H* hysteresis loop for single phase Bi(Fe_0.9_Co_0.1_)O_3_ films measured at 300 K and (**b**) Expanded view of Figure (a) (c) Different Co concentration dependence of magnetization measured at 30 and 50 kOe at 300 K for various preparation methods.

The magnetic property of single phase Bi(Fe_0.9_Co_0.1_)O_3_ epitaxial films was measured using the SQUID magnetometer at 300 K for the in-plane direction. The result is shown in Figure 5(a) and Figure 5(b). A saturation magnetization of 20 emu/cm^3^ was observed along with a weak remanent magnetization. When compared with epitaxial and polycrystalline BiFeO_3_ films, the magnetization was clearly enhanced. This magnetization might be attributed to local ferrimagnetism and/or suppression of long-range incommensurate spin cycloids and/or moderation of short-range canted *G*-type antiferromagnetism. However, further investigation of the spin structure using X-ray magnetic circular dichroism (XMCD) and neutron diffraction analysis is necessary to understand the origin of the magnetism of Bi(Fe_0.9_Co_0.1_)O_3_ films. In order to compare our results with previous works, we plotted magnetization values as a function of Co concentration. Figure 5(c) shows Co concentration of magnetization measured at 30 and 50 kOe at 300 K for various preparation methods [7,9,17]. A magnetization of 20 emu/cm^3^ at a Co concentration of 10 at.% is almost consistent with previous reports that used a different preparation method. In accordance with the magneto-electric effect in the rhombohedral structure of BiFeO_3_ [18], the Bi(Fe_0.9_Co_0.1_)O_3_ film is expected to display macroscopic magnetization changes in their electric field. We plan to study this in the future.

## 4. Conclusions

We fabricated Bi(Fe_0.9_Co_0.1_)O_3_ (50 nm) films on SrTiO_3_ (100) substrates using r.f. magnetron sputtering, while systematically changing various parameters such as Ar + O_2_ and Ar gas pressure, sputtering power, and annealing temperature to obtain single phase Bi(Fe_0.9_Co_0.1_)O_3_ films. The process window for preparing the single phase is very narrow, and the key requirement for obtaining single phase is the suppression of the formation of secondary phases of BiO*_x_*. The single phase and epitaxial structure of Bi(Fe_0.9_Co_0.1_)O_3_ films were obtained using low Ar gas pressure, low sputtering power, and an annealing temperature of 600°C in air. The epitaxial structure and homogeneity of composition were confirmed by XRD patterns and SEM-EDX, respectively. The saturation magnetization of Bi(Fe_0.9_Co_0.1_)O_3_ films increased to 20 emu/cm^3^ at RT.

## References

[1] Kubel F., Schmid H. (1993). Growth, twinning and etch figures of ferroelectric/ferroelastic dendritic BiFeO_3_ single domain crystals. J. Cryst. Growth.

[2] Wang J., Neaton J.B., Zheng H., Nagarajan V., Ogale S.B., Liu B., Viehland D., Vaithyanathan V., Schlom D.G., Waghmare U.V., Spaldin N.A., Rabe K.M., Wuttig M., Ramesh R. (2003). Epitaxial BiFeO_3_ multiferroic thin film heterostructures. Science.

[3] Lebeugle D., Colson D., Forget A., Viret M. (2007). Very large spontaneous electric polarization in BiFeO_3_ single crystals at room temperature and its evolution under cycling fields. Appl. Phy. Lett..

[4] Smolenskii G.A., Chupis I. (1982). Ferroelectromagnets. Sov. Phys. Usp..

[5] Ederer C., Spaldin N.A. (2005). Weak ferromagnetism and magnetoelectric coupling in bismuth ferrite, A. Phys. Rev. B.

[6] Azuma M.H., Kanda H., Belik A.A., Shimakawa Y., Takano M. (2007). Magnetic and structural properties of BiFe_1−x_Mn_x_O_3_. J. Magn. Magn. Mater..

[7] Naganuma H., Miura J., Okamura S. (2008). Ferroelectric, electrical and magnetic properties of Cr, Mn, Co, Ni, Cu added polycrystalline BiFeO_3_ films. Appl. Phy. Lett..

[8] Zhang Q., Kim C.H., Jang Y.H., Hwang H.J., Cho J.H. (2010). Multiferroic properties and surface potential behaviors in cobalt-doped BiFeO_3_ film. Appl. Phys. Lett..

[9] Naganuma H., Yasui S., Nishida K., Iijima T., Funakubo H., Okamura S. (2011). Enhancement of magnetization at morphotropic phase boundary in epitaxial BiCoO_3_-BiFeO_3_ solid solution films grown on SrTiO_3_ (100) substrates. J. Appl. Phys..

[10] Yasui S., Nishida K., Naganuma H., Okamura S., Iijima T., Funakubo H. (2007). Crystal structure analysis of epitaxial BiFeO_3_-BiCoO_3_ solid solution films grown by metalorganic chemical vapor deposition. Jpn. J. Appl. Phys..

[11] Nakamura Y., Kawai M., Azuma M., Shimakawa Y. (2010). Crystal structures and electric properties of (1−x)BiFeO_3_-BiCoO_3_ thin films prepared by chemical solution deposition. Jpn. J. Appl. Phys..

[12] Miyajima T., Oogane M., Kotaka Y., Yamazaki T., Tsukada M., Kataoka Y., Naganuma H., Ando Y. (2009). Direct observation of atomic ordering and interface structure in Co_2_MnSi/MgO/Co_2_MnSi magnetic tunnel junctions by high-angle annular dark-field scanning transmission electron microscopy. Appl. Phys. Exp..

[13] Naganuma H., Miura J., Nakajima M., Shima H., Okamura S., Yasui S., Funakubo H., Nishida K., Iijima T., Azuma M., Ando Y., Kamishima K., Kakizaki K., Hiratsuka N. (2008). Annealing temperature dependences of ferroelectric and magnetic properties in polycrystalline Co-substituted BiFeO_3_ films. Jpn. J. Appl. Phys..

[14] Béa H., Bibes M., Barthélémy A., Bouzehouane K., Jacquet E., Khodan A., Contour J.-P., Fusil S., Wyczisk F., Forget A., Lebeugle D., Colson D., Viret M. (2005). Influence of parasitic phases on the properties of BiFeO_3_ epitaxial thin films. Appl. Phys. Lett..

[15] Béa H., Bibes M., Fusil S., Bouzehouane K., Jacquet E., Rode K., Bencok P., Barthélémy A. (2006). Investigation on the origin of the magnetic moment of BiFeO_3_ thin films by advanced x-ray characterizations. Phy. Rev. B.

[16] Yang S.Y., Zavaliche F., Mohaddes-Ardabili L., Vaithyanathan V., Schlom D.G., Lee Y.J., Chu Y.H., Cruz M.P., Zhan Q., Zhao T., Ramesh R. (2005). Metalorganic chemical vapor deposition of lead-free ferroelectric BiFeO_3_ films for memory applications. Appl. Phys. Lett..

[17] Naganuma H., Shimura N., Miura J., Shima H., Yasui S., Nishida K., Katoda T., Iijima T., Funakubo H., Okamura S. (2008). Enhancement of ferroelectric and magnetic properties in BiFeO_3_ film by small amount of cobalt addition. J. Appl. Phys..

[18] Zhao T., Scholl A., Zavaliche F., Lee K., Barry M., Doran A., Cruz M.P., Chu Y.H., Ederer C., Spaldin N.A., Das R.R., Kim D.M., Baek S.H., Eom C.B., Ramesh R. (2006). Electrical control of antiferromagnetic domains in multiferroic BiFeO_3_ films at room temperature. Nat. Mat..

